# Theory of mind and neurocognition in early psychosis: a quasi-experimental study

**DOI:** 10.1186/s12888-014-0316-6

**Published:** 2014-12-04

**Authors:** Robyn Langdon, Michael H Connors, Megan Still, Philip B Ward, Stanley Catts

**Affiliations:** ARC Centre of Excellence in Cognition and its Disorders, and Department of Cognitive Science, Macquarie University, Sydney, NSW Australia; Rehabilitation Services, Division of Mental Health, Sydney & South Western Sydney Local Health Districts, Liverpool, NSW Australia; School of Psychiatry, University of New South Wales and Schizophrenia Research Unit, South Western Sydney Local Health District, Liverpool, NSW Australia; School of Psychiatry, University of Queensland, Brisbane, QLD Australia

**Keywords:** Early psychosis, First episode psychosis, Neurocognition, Occupational functioning, Quality of life, Schizophrenia, Social functioning, Theory of mind

## Abstract

**Background:**

People with chronic psychosis often display theory of mind impairments that are not fully accounted for by other, more general neurocognitive deficits. In these patients, both theory of mind and neurocognitive deficits contribute to poor functioning, independently of psychotic symptoms. In young people with recent-onset psychosis, however, it is unclear the extent to which theory of mind impairment is independent of neurocognitive deficits. The primary aim of this study was to examine the evidence for specific theory of mind impairments in early psychosis. A secondary aim was to explore the relations between theory of mind, neurocognition, symptom severity, and functional outcomes.

**Methods:**

Twenty-three patients who were within two years of their first psychotic episode and 19 healthy controls completed theory of mind and neurocognitive batteries. Social functioning, quality of life, and symptom severity were also assessed in patients.

**Results:**

Patients demonstrated deficits in tasks assessing theory of mind and neurocognition relative to controls. Patients’ deficits in theory of mind were evident even after adjusting for their deficits in neurocognition. Neither theory of mind nor neurocognition predicted social functioning or quality of life in this early psychosis sample. Severity of negative symptoms, however, was a significant predictor of both outcomes.

**Conclusions:**

While a specific theory of mind impairment was evident in this early psychosis sample, severity of negative symptoms emerged as the best predictor of poor functional outcome. Further early psychosis research is needed to examine the longitudinal progression of theory of mind impairments – independent of neurocognitive deficits – and their impact on psychosocial function.

## Background

Psychosis is usually associated with poor functioning. Patients with psychosis, for example, typically display poor social skills, report fewer relationships, and are evaluated more negatively in social situations than patients without psychosis [1-3]. As a result of this poor functioning, patients with psychosis experience a range of negative outcomes, including dissatisfaction with their quality of life, unemployment, and depression [1-3]. Many interventions for psychosis, therefore, focus on improving social skills and functional outcomes [4,5].

Poor functioning in psychosis may be due, in part, to a specific impairment in “theory of mind” (ToM) – the ability to ascribe mental states to others so as to predict and explain behaviour. ToM is essential to negotiating social interactions and there is strong evidence that patients across all stages of psychosis, including those with at-risk mental states, have impairment in this ability [6,7]. Indeed, social cognitive deficits, including ToM, appear to be stable over time in patients with early psychosis [8] and across patients at different stages of psychosis [7]. The poor functioning that is associated with psychosis, however, may also be due, in part, to the psychotic symptoms themselves or to other neurocognitive deficits. Patients with psychosis typically display deficits in attention, working memory, and many other aspects of neurocognition, which also predict poor functioning, independent of severity of negative symptoms [9-11].

While ToM and neurocognitive deficits appear to be present at all stages of psychosis and contribute to poor functioning, the ToM deficits – at least in chronic patients – are not fully accounted for by the other, more general neurocognitive deficits [12]. In addition, the negative effects of ToM impairment on functioning in chronic psychosis are present even when severity of other neurocognitive deficits and negative symptoms are taken into account [10]. Less is known, however, about these relations at earlier stages of psychosis. Indeed, Bora and Pantelis [6] argue that the extent to which ToM deficits are reducible to other neurocognitive deficits at earlier stages of psychosis is unclear. It is possible, for example, that the deterioration of selective theory of mind capacity and neurocognitive functioning follow different trajectories in psychosis.

To address these issues, Sullivan et al. [13] recently examined the relations between ToM, general cognition, symptoms and social functioning in a sample of patients in their first psychotic episode. Sullivan et al. found that social functioning in their sample was associated with ToM (as measured by the Hinting task) and verbal IQ, but not with other general cognitive measures or a measure of negative symptoms that excluded symptoms that overlapped with poor social functioning (e.g. asociality). These findings suggest that both theory of mind and aspects of neurocognition contribute to poor social functioning in early psychosis. However, later follow-ups found that these variables did not predict functional outcomes at six months or one year [14]. These important studies also had some limitations. The approach of excluding some negative symptoms, for example, is potentially problematic since no evidence was provided to support the categorisation of a negative symptom as social or not. In addition, Sullivan and colleagues used a rather limited neuropsychological battery. ToM was assessed with the Hinting task, for instance, which may produce skewed data that limits the analyses that can be performed. Similarly, neurocognition was assessed using only three brief measures. Given these limitations and the ambiguous findings to date, the current study sought to extend Sullivan et al.’s research using a more detailed neuropsychological battery.

### Current study

As the relations between ToM and neurocognition at early stages of psychosis have important implications for clinical interventions, we aimed to more comprehensively assess neurocognition and ToM in a first episode psychosis sample. Our primary aim was to examine theory of mind performance in early psychosis and its relative independence from neurocognition. Following previous research with patients with chronic psychosis [10], our secondary aim was to explore the relations between theory of mind, neurocognition, symptom severity, and functioning in an early psychosis sample. Patients, who were within the first two years of their first psychotic episode, and healthy controls completed batteries of ToM and neurocognition. The social functioning, quality of life, and symptom severity of patients were also assessed. If ToM was selectively impaired, as it is in people with chronic psychotic symptoms, we expected that patients and controls would show differences in this ability independent of any differences in neurocognition.

## Methods

### Participants

Twenty-three patients were recruited from two early psychosis intervention programs in New South Wales, Australia. All patients were in the first two years of their first treatment by mental health services. Patients were interviewed using the Diagnostic Interview for Psychosis [15] to confirm diagnosis according to ICD-10 criteria [16]. Seventeen of these patients had a diagnosis of “Paranoid Schizophrenia,” 4 had a diagnosis of “Undifferentiated Schizophrenia,” 1 had a diagnosis of “Schizoaffective Disorder – Bipolar Subtype,” and 1 had a diagnosis of “Other Non-Organic Psychotic Disorder.” Patients with organic brain disorders or a comorbid diagnosis of substance dependence according to the treating clinician were excluded. All the patients were prescribed low dose atypical neuroleptics but several disclosed their non-compliance, making it unreliable to explore medication effects. Consistent with the gender imbalance in young cohorts with a schizophrenia-like psychosis [17], 22 of the patients were male and one patient was female.

Nineteen healthy controls (17 male, 2 female) were recruited from the general community to match the patient group on age, gender distribution, and formal education. The controls were screened using the affective, psychotic, and substance abuse screening modules from the Structured Clinical Interview for DSM-IV Axis 1 Disorders [18]. In accord with research in this area, exclusion criteria for both groups included organic brain disorders and substance dependence (screened as above). All participants were English-speaking and gave written informed consent. Demographic features of both groups and clinical features of patients are summarised in Table 1.

**Table 1:** **Demographics of patients and controls**

	**Patients**	**Healthy controls**	**Significance test**
Males:females	22:1	17:2	χ^2^(1) = .599
Age (years)	20.91 ± 1.83 (18-25)	20.79 ± 1.81 (17-24)	*t*(40) = .219
Education (years)	11.43 ± 2.02 (8-18)	12.82 ± 1.94 (9-16)	*t*(40) = 2.247*
IQ	96.65 ± 8.41	103.42 ± 9.32	*t*(40) = 2.472*
Age of illness onset (years)	19.91 ± 1.95 (16-24)		
Duration of illness (weeks)	50.74 ± 29.50 (12-104)		
SAPS Positive Symptoms	1.25 ± .94 (0.00-3.75)		
SANS Negative Symptoms	2.18 ± .72 (0.60-3.80)		
Social Functioning	50.87 ± 12.12 (30-80)		
Quality of Life	58.22 ± 22.78 (20-120)		

*Note.* Data expressed as means ± SD (range in parentheses). *p < .05. Positive and negative symptoms assessed using the Scales for the Assessment of Positive and Negative Symptoms of Schizophrenia (SAPS and SANS: Andreasen, 1983, 1984). The overall Positive and Negative rating is the average of global ratings on the SAPS and SANS respectively (‘0’ = absent; ‘1’ = questionable; ‘2’ = mild; ‘3’ = moderate; ‘4’ = marked; ‘5’ = severe).

The study followed the *World Medical Association Declaration of Helsinki – Ethical Principles for Medical Research Involving Human Subjects*, and was approved by the Hunter Area Research Ethics Committee (reference number 01/12/12/3.23), South Western Sydney Area Health Service Research Ethics Committee (reference number 00/082), and the University of Sydney Human Ethics Committee (reference number 00/10/03).

### Materials and procedure

#### Theory of Mind (ToM)

The first ToM measure was a non-verbal picture-sequencing task [19-21]. Participants were shown four picture-cards in a fixed, incorrect order. Participants were asked to reorder the picture-cards to provide a logical sequence of events. There were four types of sequences (four sequences per type): ToM “false belief stories” that required participants to go beyond the immediate objective information to infer a character’s mistaken belief; “social-script stories” that controlled for simple social reasoning; “mechanical stories” that controlled for physical cause-and-effect reasoning; and “capture stories” that controlled for inhibition of an obvious but misleading cue. Each sequence scored two points if the first card was positioned correctly, two points if the last card was positioned correctly, and one point each if the second and third cards were positioned correctly. Scores were averaged across each type of story (range 0–6).

The second ToM measure was a joke appreciation task [20-22]. Participants were shown a series of visual cartoons and asked to explain the humour. There were 11 “ToM” cartoons in which the joke depended on understanding a character’s false belief or mental state, and 11 “control” cartoons in which the joke did not depend on inferring mental states but instead involved situational anomalies. Responses were scored from 0 (an incorrect or irrelevant answer) to 3 (a complete, correct explanation). Scores were averaged across the two types of cartoon.

The third ToM measure was a story comprehension task [20-22]. Participants were asked to read a series of stories and answer a question about each. There were eight “ToM” stories that involved understanding the mental states of the characters and eight control stories that required only general comprehension. The length of the stories and the complexities of the sentences were matched across the two types of story. Responses were scored from 0 (an incorrect answer) to 2 (a complete, correct answer). Scores were averaged across the two types of story.

#### Neurocognition

Participants also completed a battery of neurocognition tests. IQ was estimated using the National Adult Reading Test [23]. Visual memory was assessed using the visual memory span test from the Wechsler Adult Intelligence Scale-Revised [24]. Verbal memory was assessed using the logical memories subtest from the WAIS [24]. Planning was assessed using the number of planning moves on a computerised Tower of London task [25]. Set shifting was assessed using the number of categories achieved on the Wisconsin Card Sort Test [26]. Verbal fluency was assessed using the Controlled Oral Word Association Test [27]. Semantic fluency was assessed by asking participants to generate the names of as many exemplars of a category (in this study; food, animals, and furniture) that they could think of in 60 seconds. Inhibition was indexed by time taken on the colour-word interference condition that followed the colour-naming and colour-word reading conditions of a bespoke Stroop task [28].

#### Clinical interviews

The scales for Assessment of Positive and Negative Symptoms of Schizophrenia [29,30] were used to rate symptom severity in patients. The social and global functioning of patients were also assessed using the Social and Occupational Functioning Assessment Scale [31] and the Quality of Life Scale [32].

### Statistical analyses

The correlations between scores from the three ToM tasks were examined first to assess convergent validity. To obtain a global index of ToM abilities, scores from the three ToM measures were then converted to z-scores and averaged to produce a single composite ToM score.

ToM and neurocognition scores of the patients and controls were compared using independent samples t-tests. ToM and neurocognitive score differences between patients and controls were converted to effect sizes in terms of Cohen’s d. In order to further reduce the data for subsequent analyses, a composite neurocognition score was also calculated by converting scores from the individual measures into z-scores across groups, scaled such that higher scores indicated better function, and averaged to produce a single composite neurocognition score. An ANCOVA was then used to compare ToM between groups, adjusting for the composite score of neurocognition.

To explore the relations between ToM, neurocognition, symptom severity, and functioning, zero-order correlations were assessed between these measures in patients. To examine predictors of functioning, hierarchical regression analyses were conducted with social functioning and quality of life as the dependent variables, and neurocognition, ToM and severity of negative symptoms entered as the predictors.

## Results

### Relationships between ToM measures

All three measures of ToM correlated with each other. The picture sequencing ToM score correlated with ToM scores from both the joke appreciation task, *r*(42) = .52, *p* < .01, and the story comprehension task, *r*(42) = .47, *p* < .01. The latter two measures also correlated significantly, *r*(42) = .59, *p* < .01.

### Specificity of ToM deficits in patients

**Figure 1:**
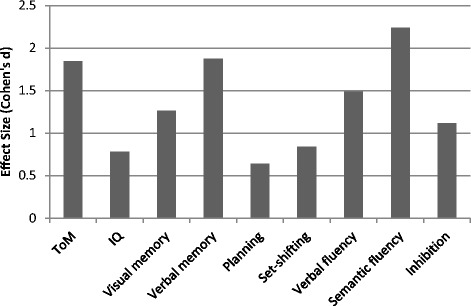
**Patients’ deficits in ToM and different neurocognitive domains relative to controls.**

Patients performed worse than controls in ToM tasks and in all measures of neurocognition. These differences, however, did not reach statistical significance for set shifting and planning (see Table 2). Effect sizes are shown in Figure 1. Patients showed particularly large impairments in ToM, verbal memory, and semantic fluency relative to controls. To determine whether ToM deficits were evident when neurocognitive performance was accounted for, we compared patients and controls’ composite ToM scores using an ANCOVA with the composite score of neurocognition as a covariate. Patients still displayed a significant deficit in ToM compared to controls, *F*(1, 38) = 5.60, *p* = .02, η_p_^2^ = .13. There was also a significant effect of neurocognition on ToM independent of group, *F*(1, 38) = 25.81, *p* < .01, η_p_^2^ = .41. Levene’s test indicated that the assumption of equality of error variances was met for this analysis, *F*(1, 39) = 3.83, *p* = .06. Patients and controls did not differ on any of the control conditions in the ToM tasks (all *p*s > .05).

**Table 2:** **Differences between patients and controls in ToM and neurocognition**

	**Patients**	**Healthy controls**	**Significance test:** ***t*** **(40)**
ToM	−1.60 ± 2.16	1.94 ± 1.04	6.55**
Picture sequencing	4.80 ± 1.19	5.87 ± .28	3.81**
Joke appreciation	1.26 ± .47	1.90 ± .27	5.23**
Story comprehension	.72 ± .31	1.19 ± .27	5.17**
Neurocognition	−3.66 ± 3.77	4.21 ± 3.89	6.57**
IQ	96.65 ± 8.41	103.42 ± 9.32	2.47*
Visual memory	16.78 ± 3.26	19.84 ± 2.73	3.25**
Verbal memory	26.39 ± 15.76	54.21 ± 13.44	6.08**
Verbal fluency	28.00 ± 9.29	44.58 ± 13.47	4.71**
Semantic fluency	32.43 ± 9.45	56.95 ± 12.94	7.09**
Inhibition	32.30 ± 9.88	23.24 ± 5.92	3.49**
Set-Shifting	-.17 ± .93	.20 ± .50	1.54
Planning	60.26 ± 7.84	55.63 ± 6.79	2.02

*Note.* Data expressed as means ± SD. *p < .05, **p < .01.

### ToM, neurocognition, symptom severity, and functioning in patients

Correlations between ToM, neurocognition, symptom severity, and functional outcomes are shown in Table 3. Focusing on correlations with the latter, ToM did not correlate significantly with either quality of life or the SOFAS social functioning measure. Neurocognition was significantly correlated with quality of life. This relationship, however, was not significant in a partial correlation that controlled for negative symptoms (*r*_p_ = .70, *p* = .76). Negative symptoms were associated with poorer social functioning and quality of life, as well as lower ToM and neurocognition. Positive symptoms were not significantly correlated with any of the other domains assessed (all *p*s > .42).

**Table 3:** **Zero-order correlations between ToM, neurocognition, symptom severity, and social functioning in patients**

	**ToM**	**Neurocognition**	**Negative symptoms**	**Positive symptoms**	**Quality of life**	**Social functioning**
ToM		.70**	-.50*	-.01	.26	.16
Neurocognition	.70**		-.47*	−.06	.53*	.30
Negative symptoms	-.50*	-.47*		.18	-.50*	-.51*
Positive symptoms	-.01	-.06	.18		.02	-.09
Quality of Life	.26	.53*	-.50*	.02		.85**
Social Functioning	.16	.30	-.51*	-.09	.85**	

*Note.* *p < .05, **p < .01.

### Predictors of social functioning and quality of life

Since previous research indicates that neurocognitive deficits contribute to theory of mind impairment which, in turn, associates with negative symptoms (in accord with our zero-order correlation results), we conducted follow-up hierarchical regression analyses to examine the cumulative effects of these measures when predicting patients’ social functioning and quality of life. Table 4 summarises the results. While acknowledging the need for caution given our small sample size and the intercorrelations between predictors, findings indicate that neither neurocognition, nor the combination of neurocognition and ToM, predicted our functioning measures in this early psychosis sample. In contrast, severity of negative symptoms was a significant predictor of poor social functioning and showed a similar trend for reduced quality of life, having adjusted for the effects of neurocognition and ToM. Subsequent backward regression analyses reduced the full models in each case to leave negative symptoms as the sole predictor, *F*(1,20) = 7.37, *p* = .01 and *F*(2,19) = 6.25, *p* = .01 respectively.

**Table 4:** **Summary of hierarchical regression analyses predicting social functioning and quality of life in early psychosis patients**

	**ß**	**t**	***p***	**R** ^**2**^
*Predicting SOFAS Scores of Social Functioning*				
**Step 1**				.09
Neurocognition	.30	1.39	.18	
**Step 2**				.09
Neurocognition	.34	1.12	.28	
ToM	-.07	.21	.83	
**Step 3**				.30
Neurocognition	.20	.70	.49	
ToM	-.22	.76	.46	
Negative symptoms	-.53	2.30	.03*	
*Predicting QLS Scores of Life Satisfaction*				
**Step 1**				.29
Neurocognition	.53	2.83	.01	
**Step 2**				.29
Neurocognition	.60	2.22	.04	
ToM	-.09	.33	.74	
**Step 3**				.42
Neurocognition	.48	1.89	.08	
ToM	-.21	.81	.43	
Negative symptoms	-.42	2.00	.06^!^	

*p < .05; ^!^ *p* < .10.

## Discussion

Consistent with previous research [6], patients in the early stages of psychosis demonstrated deficits in both ToM and neurocognition. Importantly, however, while ToM deficits co-occurred with deficits in neurocognition – particularly deficits in verbal memory and semantic fluency – they were not fully accounted for by these neurocognitive deficits. This evidence for specificity of ToM impairment at early stages of psychosis is consistent with evidence elsewhere that ToM deficits may be a trait marker of schizophrenia [6,25].

Nevertheless, neither ToM nor neurocognitive deficits in the early psychosis patients predicted poor functional outcomes. This is consistent with Sullivan et al.’s [14] finding that ToM does not predict poor outcomes longitudinally in the early stages of psychosis. The findings, however, are in contrast to other cross-sectional research which has found that ToM deficits are associated with poor social functioning in both early [13] and chronic psychosis [10]. These divergent findings may reflect differences in methodology. The current study used different measures of ToM to those employed in Sullivan et al.’s [13] cross-sectional study, which used the Hinting task to test sensitivity to intended meanings of indirect hints. It is possible that the Hinting task taps social knowledge in addition to ToM and so better predicts functional outcomes. Sullivan et al.’s [14] failure, however, to find an association longitudinally, together with the current findings, suggests that other factors, including severity of negative symptoms, may play a greater role in determining real-world functioning, at least in the early stages of psychosis.

The current study was limited by its relatively small sample size and the fact that it did not consider the effects of general psychopathology and comorbid Axis-II symptomatology. With regard to the latter, borderline traits in adolescents are associated with ‘hyper-mentalising’ errors (i.e., errors of inferring mental states in others without reasonable justification) [33], rather than the ‘hypo-mentalising’ errors (i.e., errors of failing to infer mental states) seen in our early psychosis sample. In addition, we could not examine medication effects and relied on the SOFAS and QLS, which although widely-used, are based on interview and provide only gross global estimates of functioning. Future research could use more sensitive and targeted measures of functioning, including scales that require direct observation of actual or role-played functioning, to assess different domains of functional outcomes. Future research could also examine whether subtypes of negative symptoms differentially impact functioning [34].

In conclusion, patients in our early psychosis sample displayed deficits in ToM, which were independent of their neurocognitive deficits. Negative symptoms, and not these cognitive deficits, were the strongest predictor of poor functioning. This is in contrast with the evidence that ToM deficits are the strongest predictors of social dysfunction in patients living with chronic psychotic symptoms [10]. In addition, the significant correlations in our sample between negative symptoms and impaired ToM and neurocognition indicate the challenges of separating their effects on functioning. While our results require replication with a larger, more gender-balanced early psychosis sample, they reinforce the need for future early psychosis research to examine the developmental progression of ToM deficits and their real-world consequences over time.
